# Supplementary material for: The impact of Astragaloside IV on the inflammatory response and gut microbiota in cases of acute lung injury is examined through the utilization of the PI3K/AKT/mTOR pathway
Source: PLoS One. 2024 Jul 2;19(7):e0305058. doi: 10.1371/journal.pone.0305058 (PMC11218977; doi:10.1371/journal.pone.0305058)
Supplement: S1 Graphical abstract — (DOCX) [file pone.0305058.s001.docx]

Graphic abstract
