# Supplementary figures and images for: The impact of Astragaloside IV on the inflammatory response and gut microbiota in cases of acute lung injury is examined through the utilization of the PI3K/AKT/mTOR pathway
Source: PLoS One. 2024 Jul 2;19(7):e0305058. doi: 10.1371/journal.pone.0305058 (PMC11218977; doi:10.1371/journal.pone.0305058)

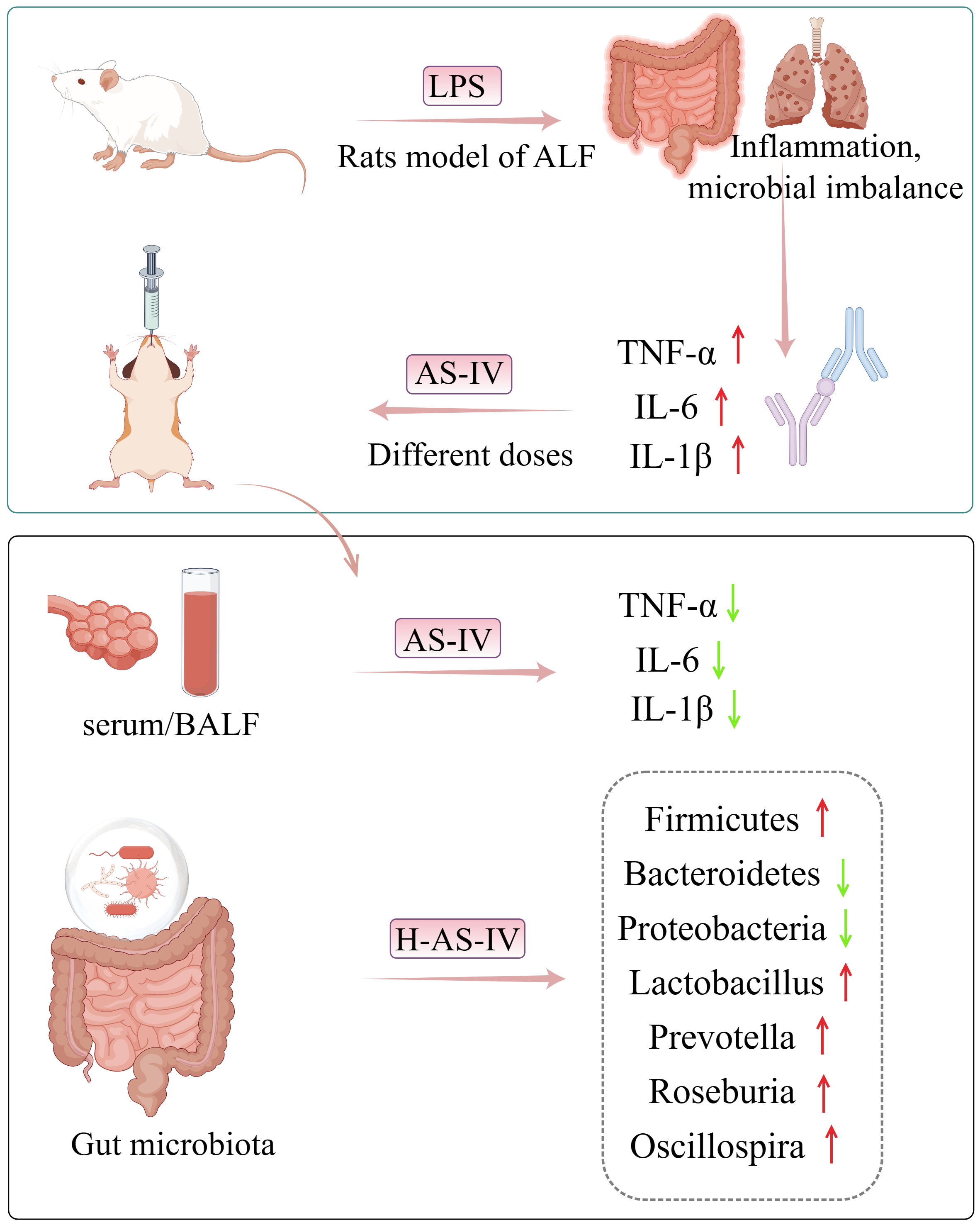

Supplement: S2 Graphical abstract — (TIFF) [file pone.0305058.s002.tiff]

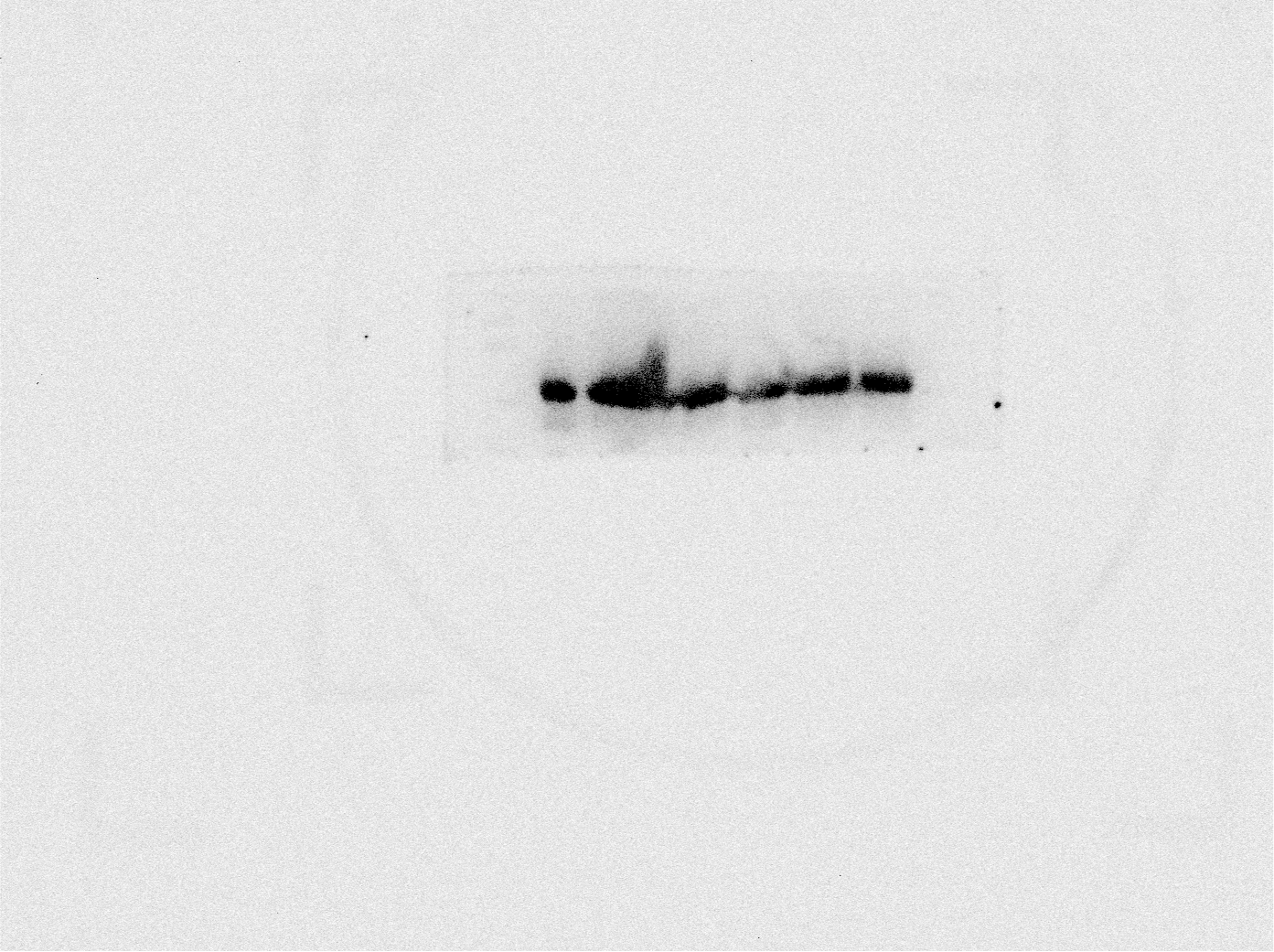

Supplement: S1 Data — (ZIP) [file pone.0305058.s004.zip › minimal data set/AKT.tif]

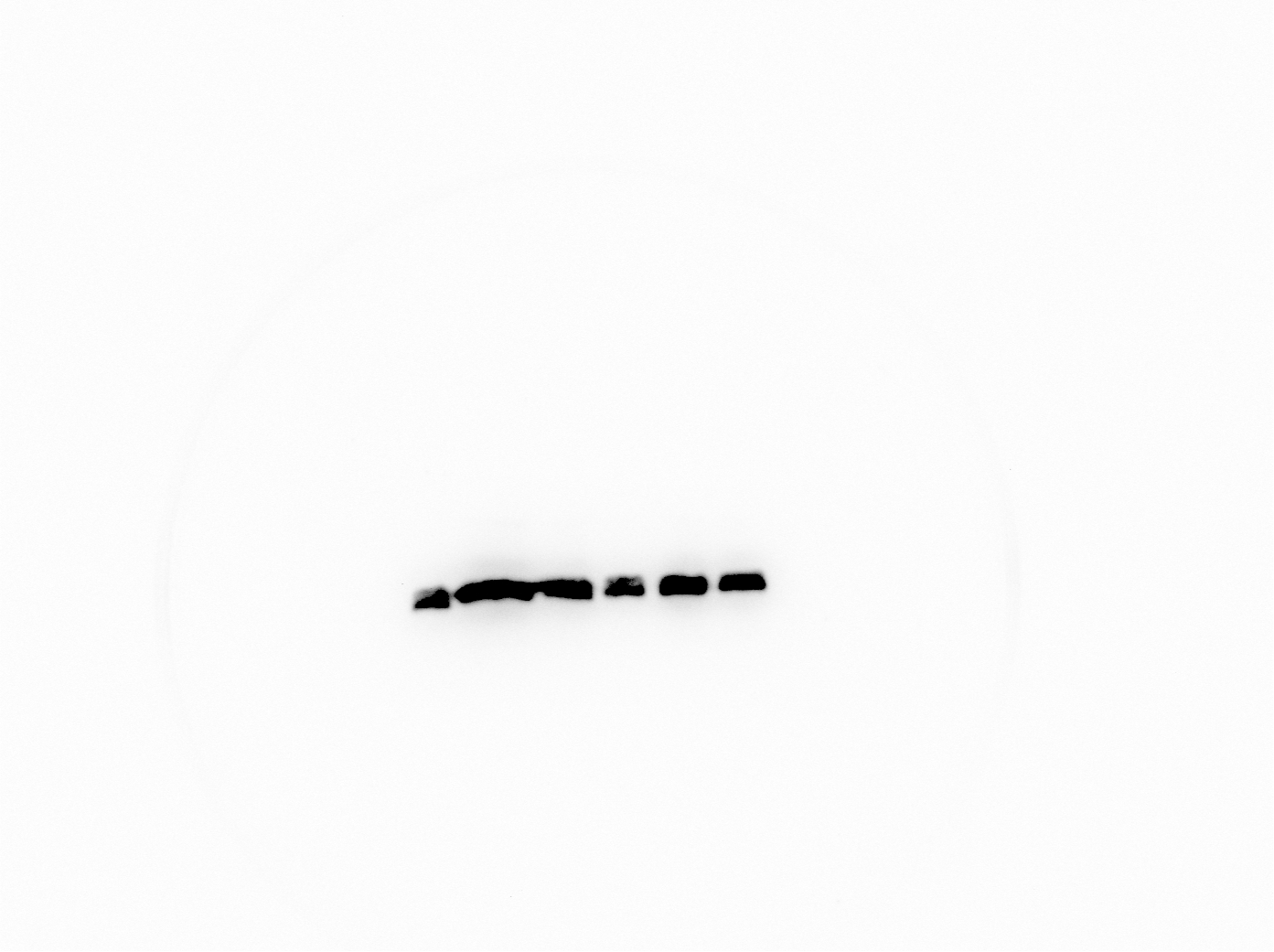

Supplement: S1 Data — (ZIP) [file pone.0305058.s004.zip › minimal data set/mTOR.tif]

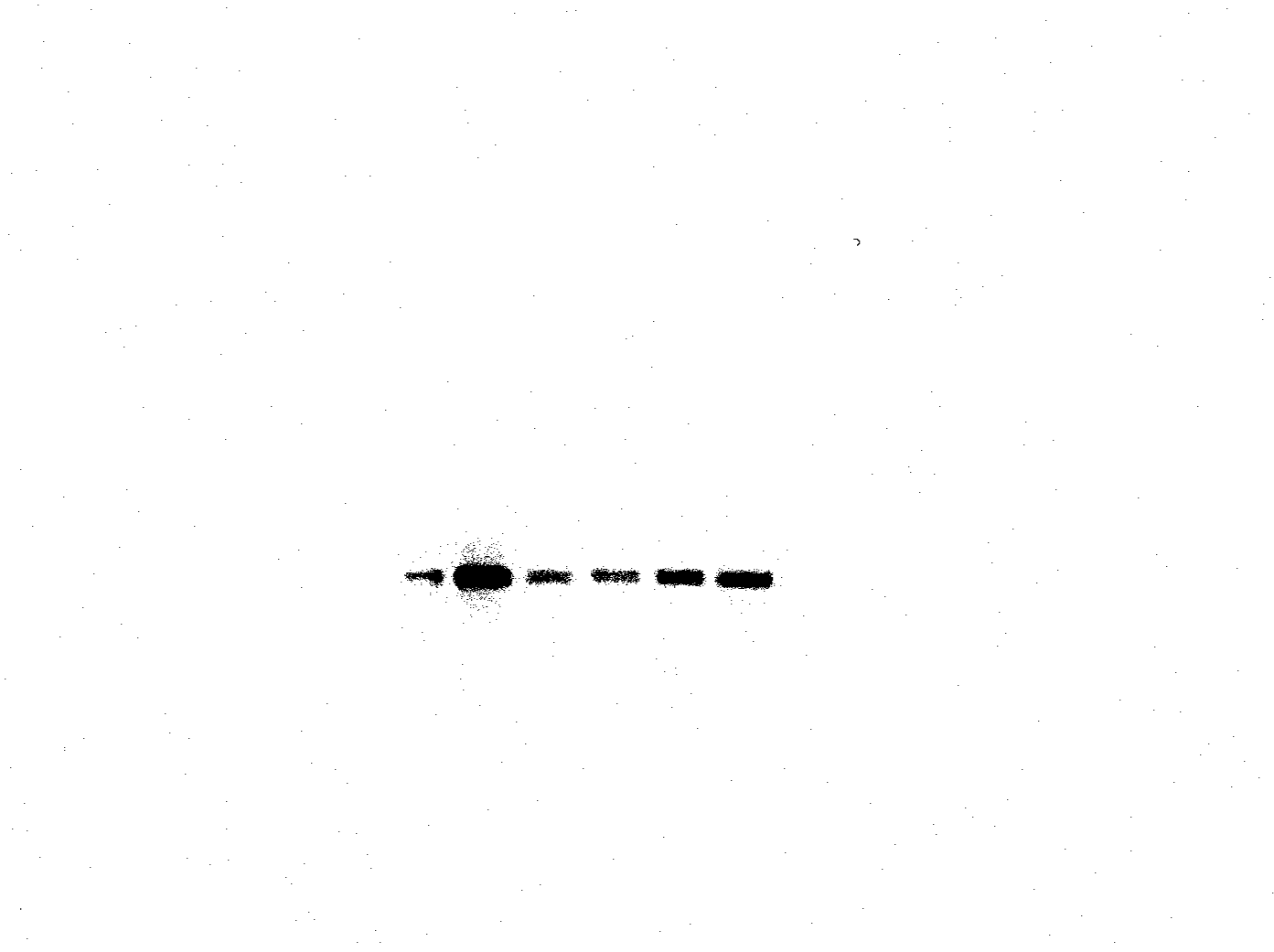

Supplement: S1 Data — (ZIP) [file pone.0305058.s004.zip › minimal data set/pAKT.tif]

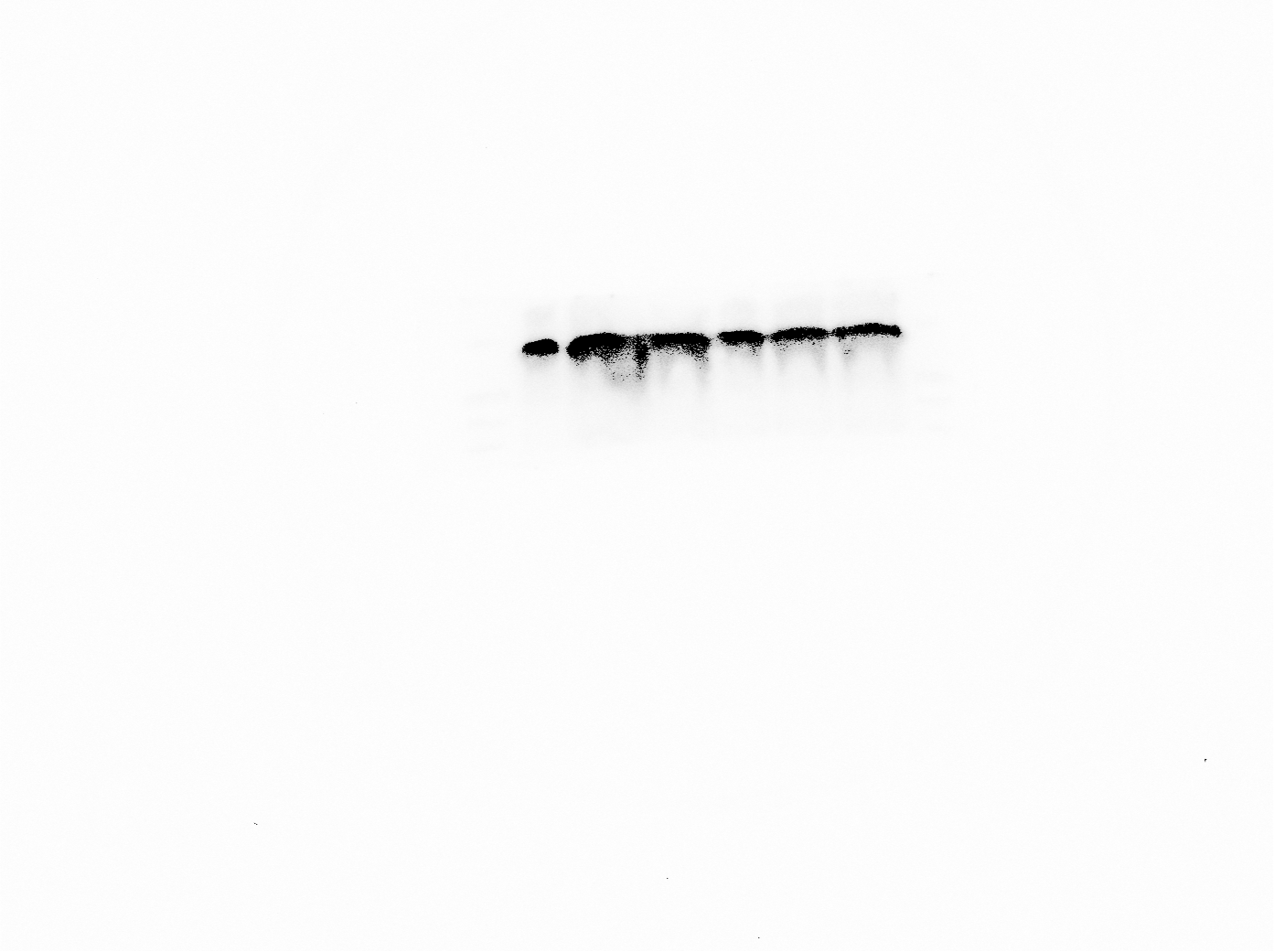

Supplement: S1 Data — (ZIP) [file pone.0305058.s004.zip › minimal data set/PI3K.tif]

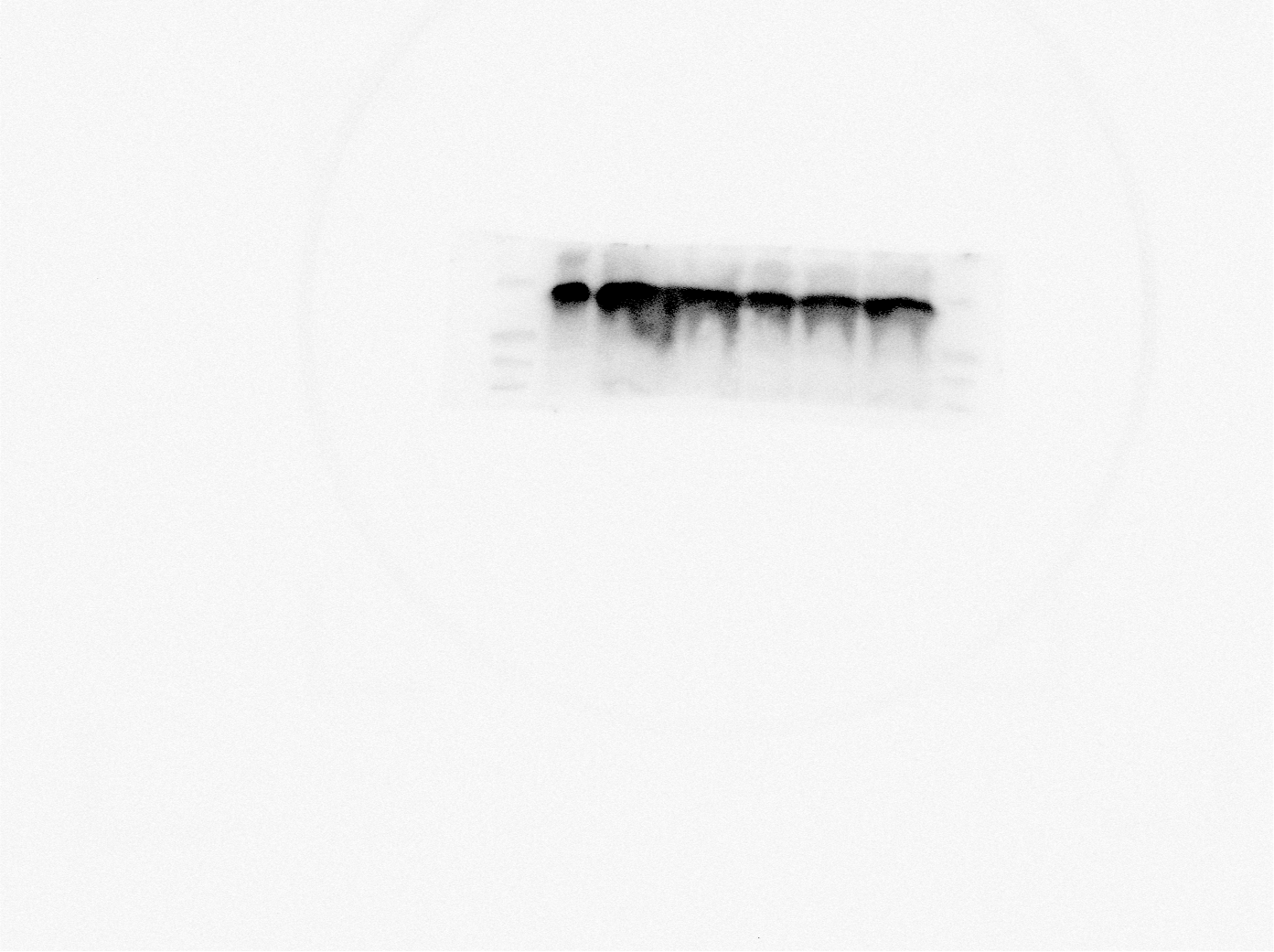

Supplement: S1 Data — (ZIP) [file pone.0305058.s004.zip › minimal data set/pmTOR.tif]

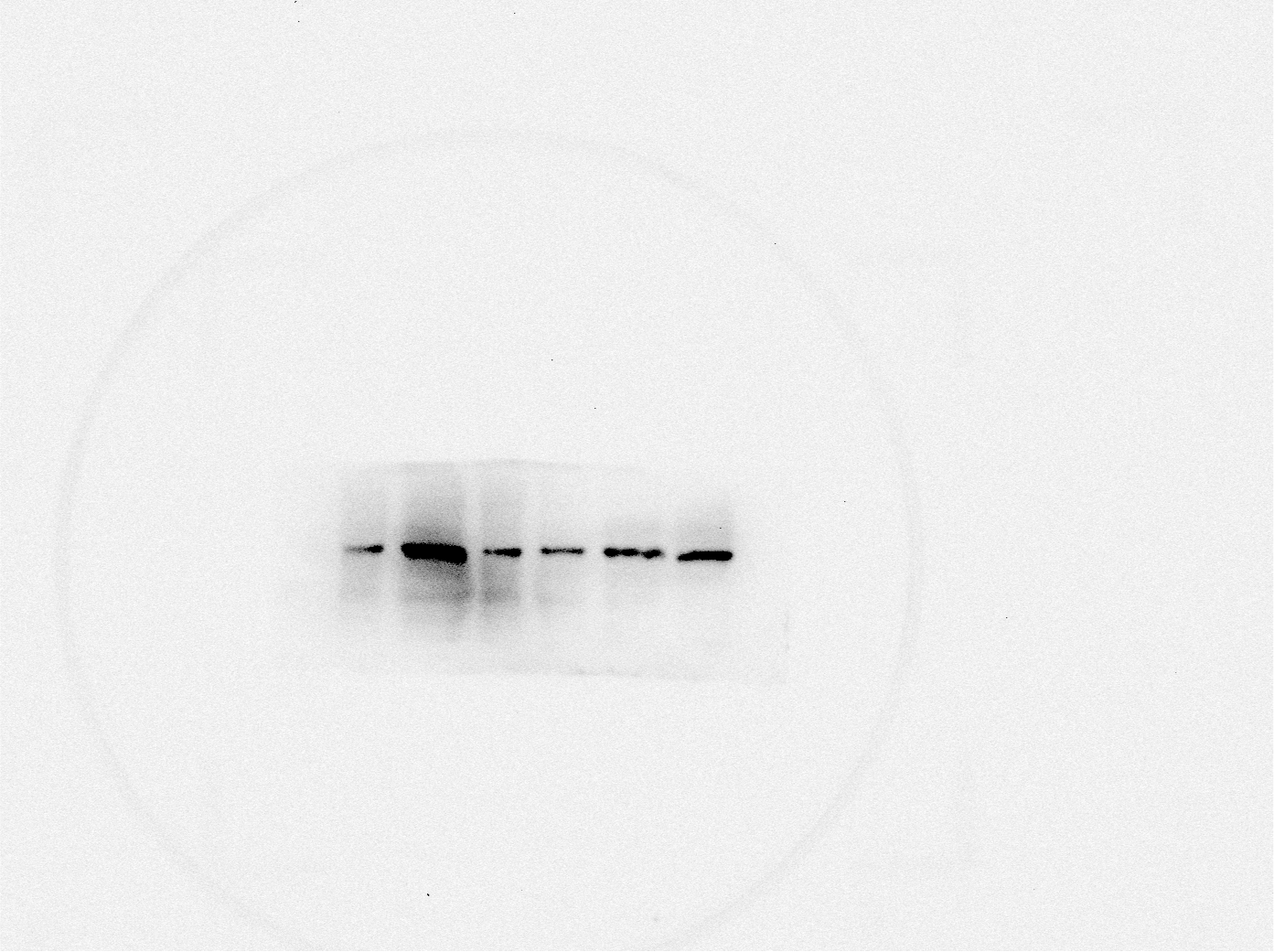

Supplement: S1 Data — (ZIP) [file pone.0305058.s004.zip › minimal data set/pPI3K.tif]

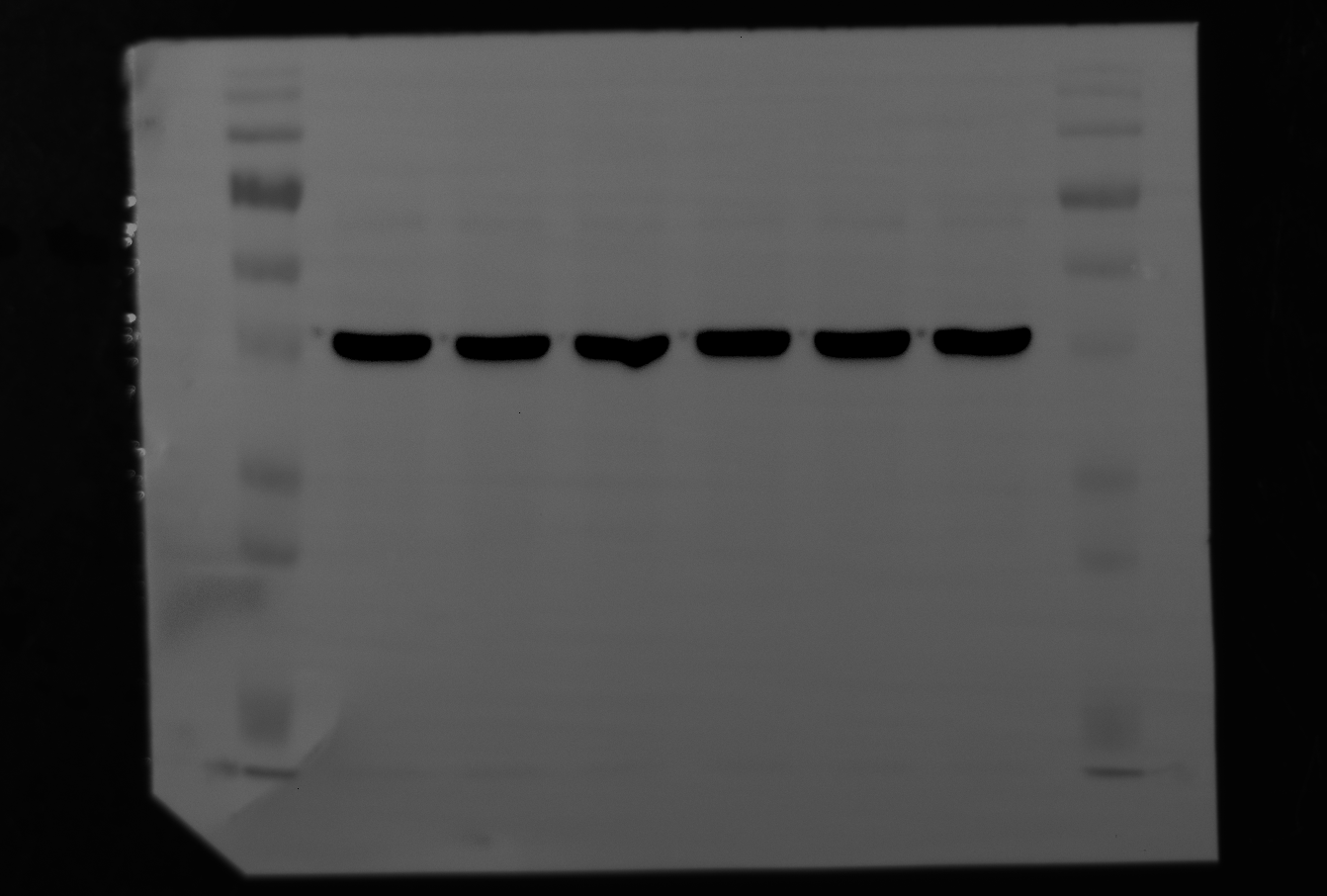

Supplement: S1 Data — (ZIP) [file pone.0305058.s004.zip › minimal data set/β-ACTIN.tif]
