# Supplementary material for: The impact of Astragaloside IV on the inflammatory response and gut microbiota in cases of acute lung injury is examined through the utilization of the PI3K/AKT/mTOR pathway
Source: PLoS One. 2024 Jul 2;19(7):e0305058. doi: 10.1371/journal.pone.0305058 (PMC11218977; doi:10.1371/journal.pone.0305058)
Supplement: S1 Raw images — (DOCX) [file pone.0305058.s003.docx]

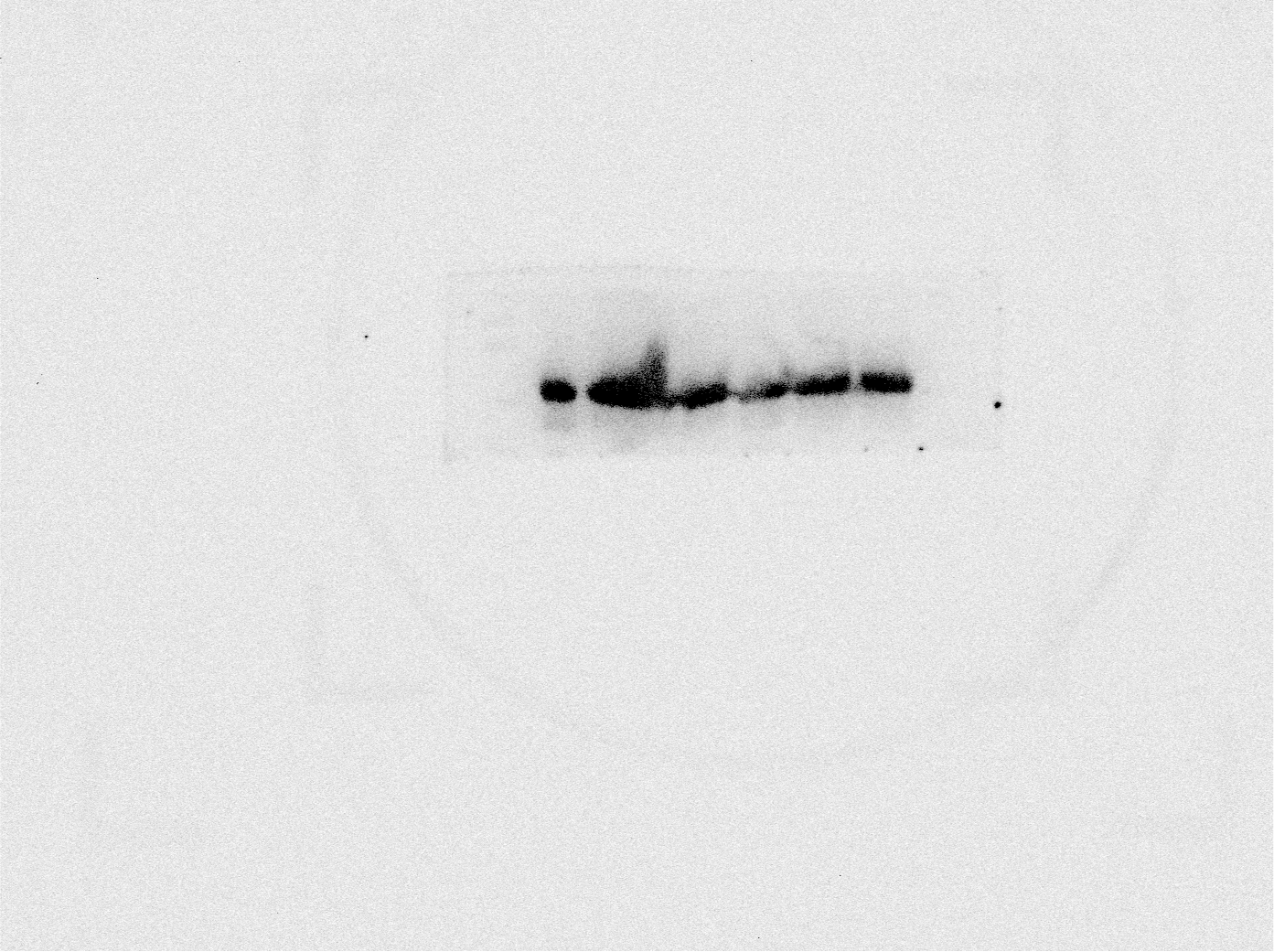


Fig 8. original image of AKT


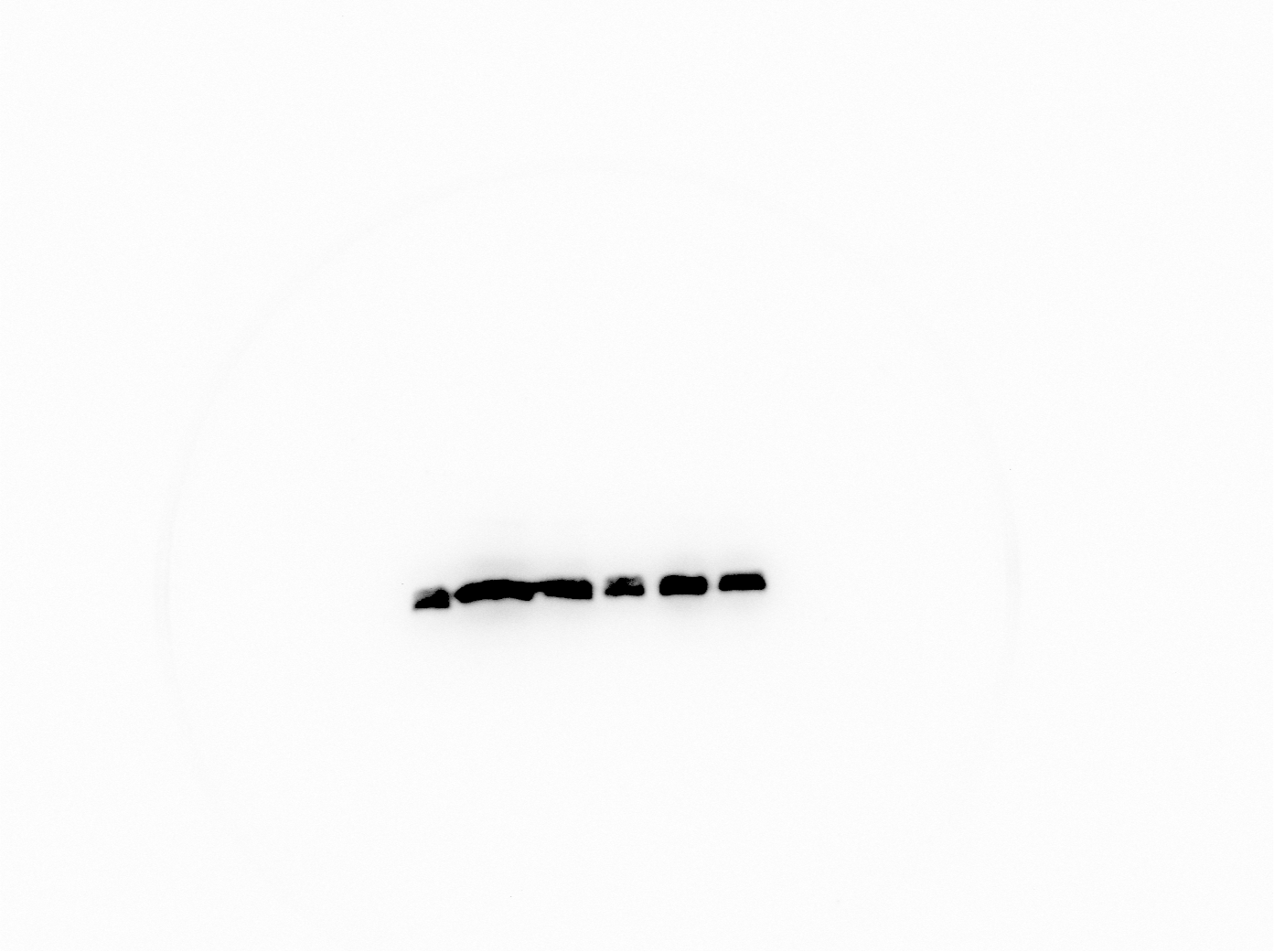


Fig 9. original image of mTOR


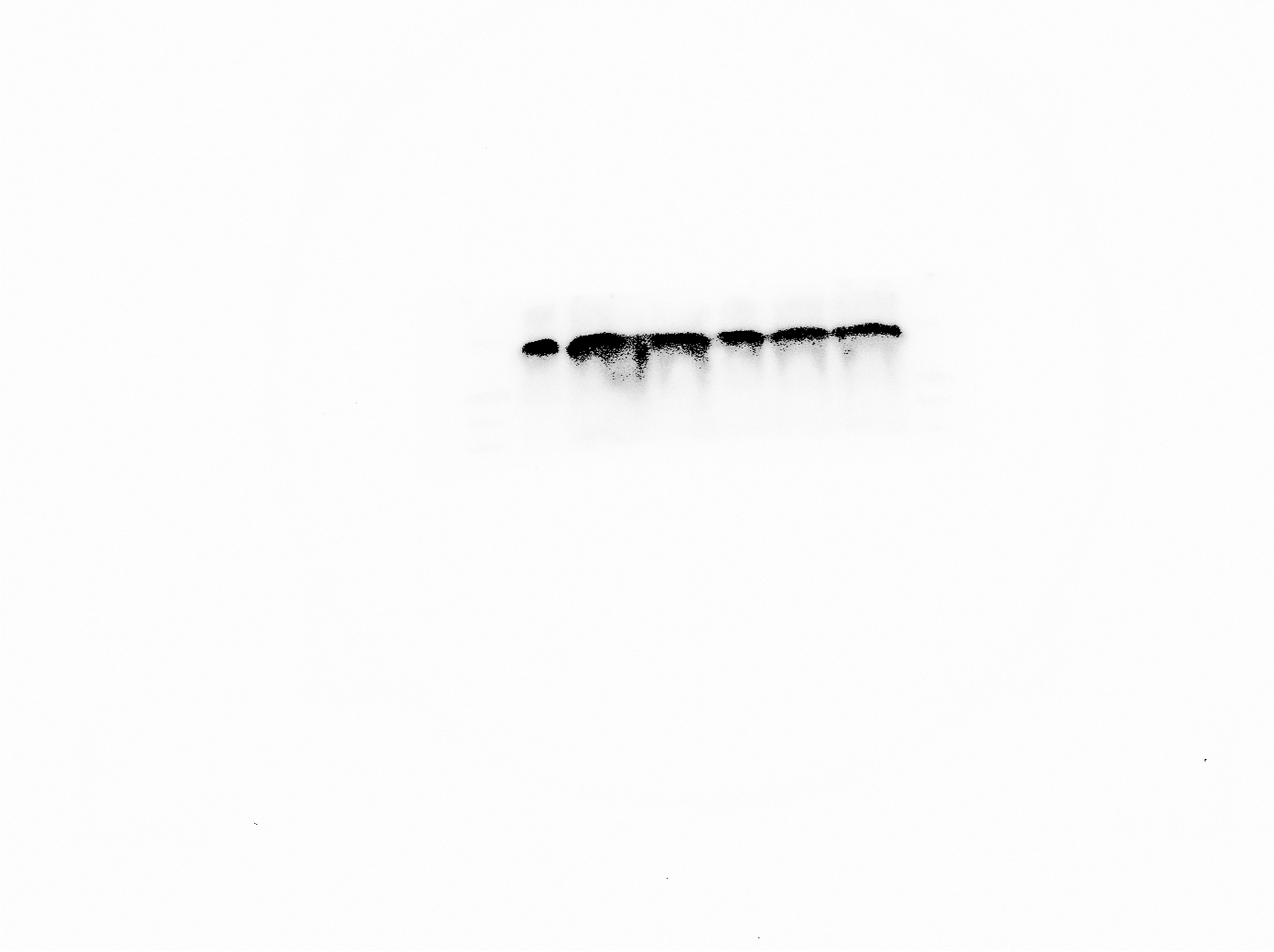


Fig 9. original image of PI3K


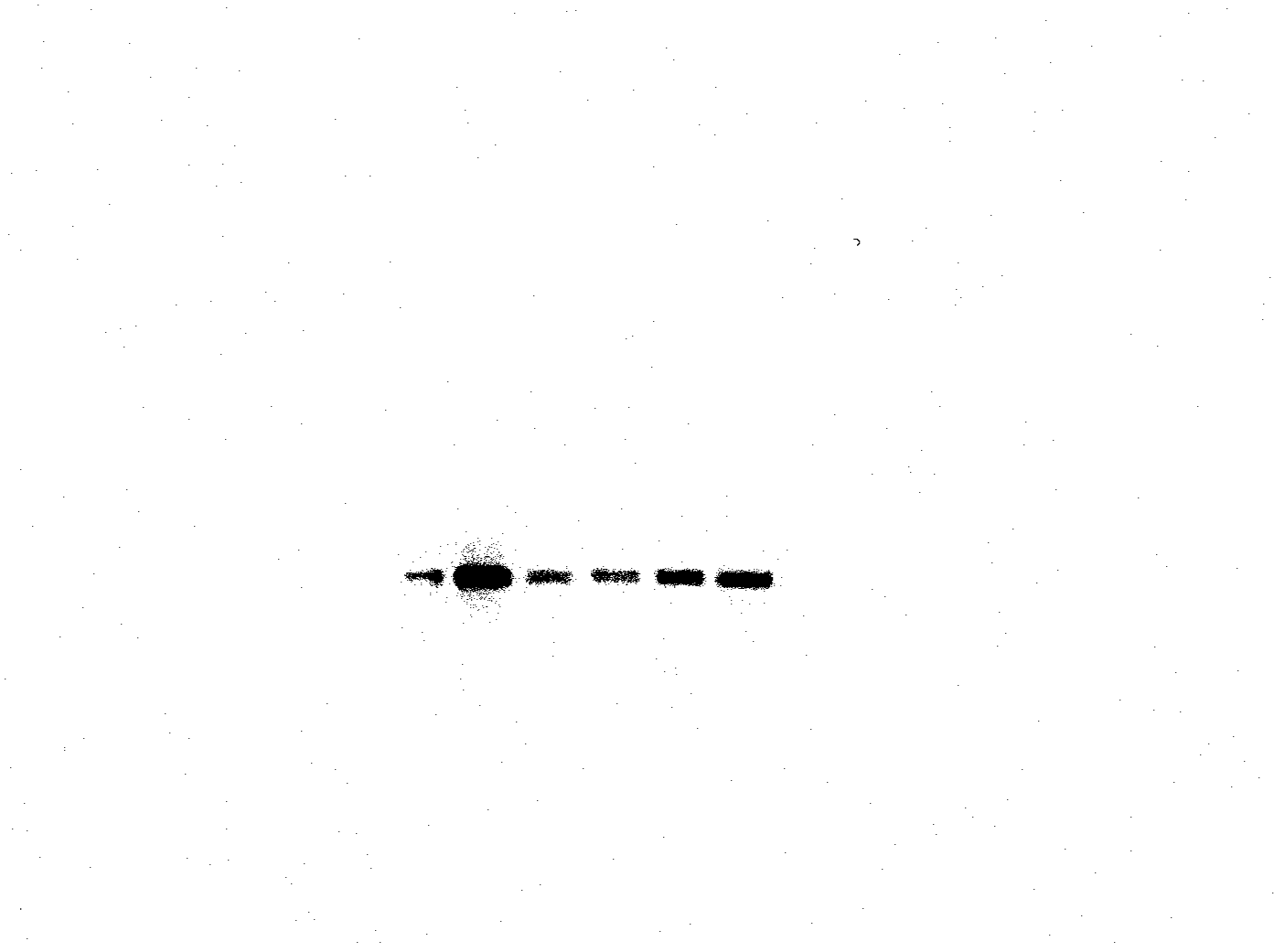


Fig 10. original image of p-AKT


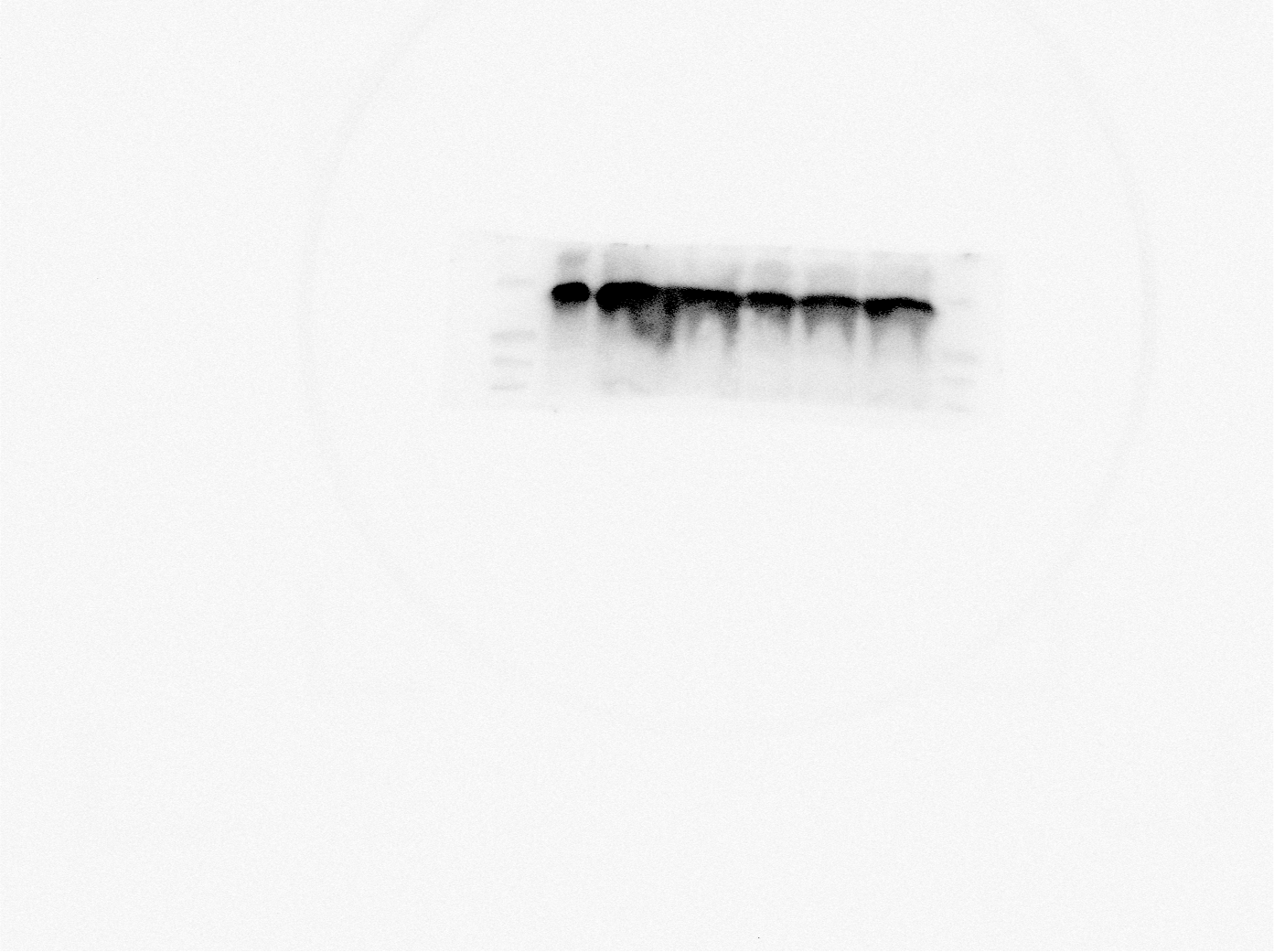


Fig 11. original image of p-mTOR


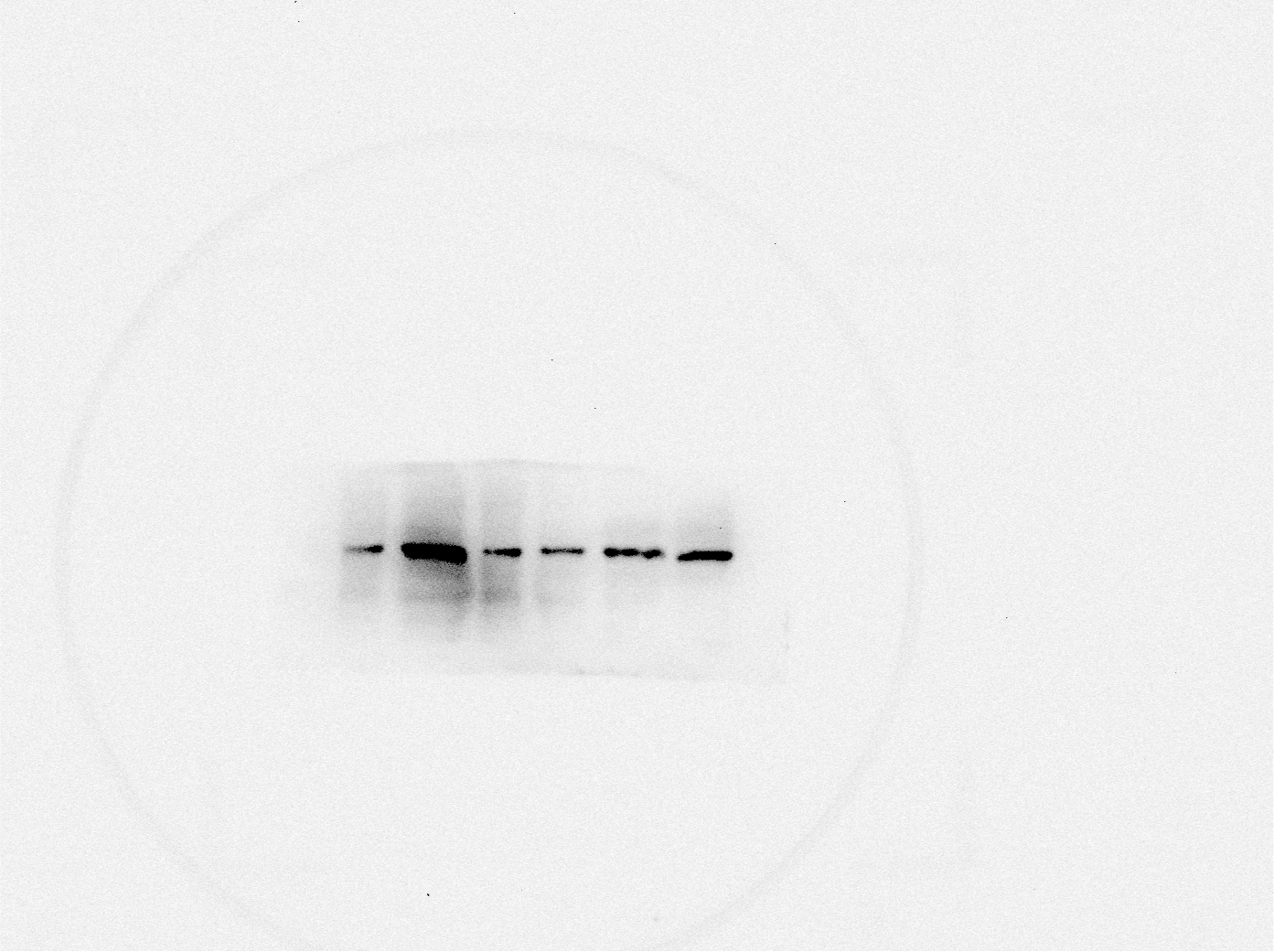


Fig 12. original image of p-PI3K


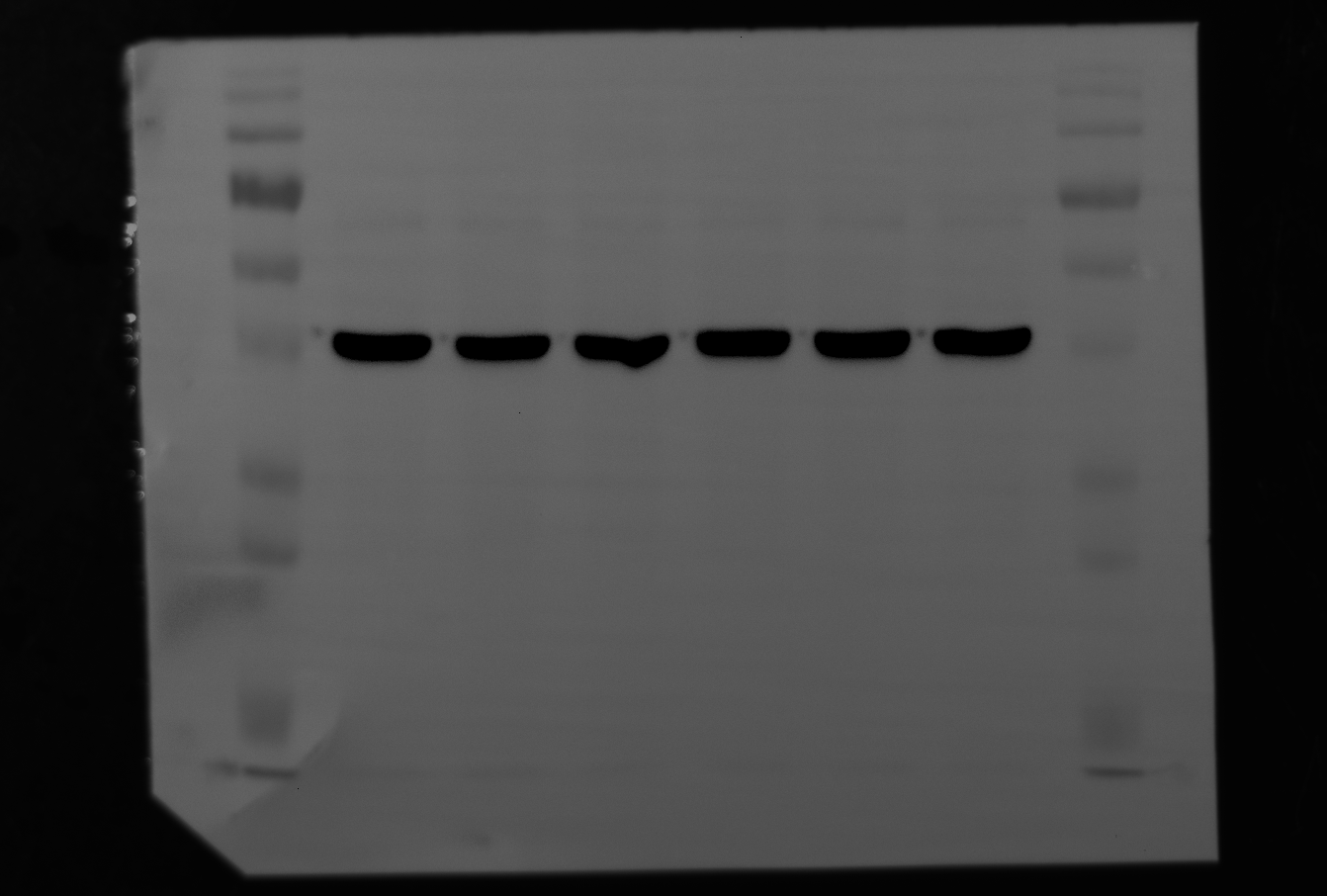


Fig 13. original image of βACTIN
